# Book on the War

**Published:** 1862-11

**Authors:** 


BOOK ON THE WAR—*The History of the Civil War in America*, comprising a full and impartial account of the origin and progress of the rebellion; of the various naval and military engagements; of the heroic deeds performed by armies and individuals, and of touching scenes in the field, the camp, the hospital, and in the cabin, by J. S. C. ABBOTT, author of the "Life of Napoleon," "History of the French Revolution," "Monarchies of Continental Europe," &c., &c. Illustrated with maps, diagrams and numerous steel engravings of battle scenes from original designs, by Darley and other eminent artists, and portraits of distinguished men, in two volumes. Vol. 1, containing over 450 pages, is to be delivered in a few weeks. We have seen specimens of this work, and think it must meet with extensive encouragement, as every one is highly interested in the matter it contains. The illustrations and portraits of distinguished men are worth vastly more than the cost of the book. To the student and the politician it will be invaluable as a work of reference, and of all the histories of this war, now being written, will be every way the most desirable.

